# The Role of Inflammatory Mediators in Immune-to-Brain Communication during Health and Disease

**DOI:** 10.1155/2013/429231

**Published:** 2013-09-10

**Authors:** Diego Gomez-Nicola, Jessica Teeling, Carmen Guaza, Jonathan P. Godbout, Dennis D. Taub

**Affiliations:** ^1^Centre for Biological Sciences, University of Southampton, Southampton SO16 6YD, UK; ^2^Cajal Institute, CSIC, 28003 Madrid, Spain; ^3^Department of Neuroscience, Center for Brain and Spinal Cord Repair, Columbus, OH 43210, USA; ^4^Hematology and Immunology Research Section, Medical Services, VA Medical Center, Department of Veteran Affairs, Washington, DC 20422, USA

The field of neuroimmunology is providing growing evidence of active crosstalk between the immune system and the nervous system in health and during diverse pathological conditions. Experimental and clinical research now suggests that signaling from periphery to brain is important for maintaining homeostasis, but also has the potential to impact brain disease initiation or progression. Knowledge and understanding of the cellular and molecular mechanisms governing immune-to-brain bidirectional communication is providing key insights to better model neuroimmune communication, understand the clinical implications, and design better therapies for CNS disorders with an inflammatory component.

This special issue contains 16 papers, representing original research articles, clinical research articles, and reviews, ranging from detailed studies of the key molecular determinants of the neuroinflammatory reaction to the complex neuroimmune component of diverse neurological diseases.

*“Systemic immune activation leads to neuroinflammation and sickness behavior in mice”* by S. Biesmans et al. describes a study that investigates the effect of systemic LPS administration on the CNS by assessing changes of glial cells, cytokine production, and various behavioural tasks. The use of GFAP-luc mice provides novel insight into the glial response to systemic inflammation. The study also provides insights into the kinetics of behavioural changes in response to systemic inflammation, when measured in the same subject. *“Immune privilege as an intrinsic CNS property: astrocytes protect the CNS against T-cell-mediated neuroinflammation” *by U. Gimsa et al. is a review of the literature on neuroimmune interactions between CNS astrocytes and peripheral T cells that attempt to gain access into the CNS. This review provides evidence of these astrocyte-T cell interactions in both health and disease. The authors discuss mechanisms by which astrocytes regulate T cells to help direct both pro- and anti-inflammatory responses. Overall, the authors review the evidence that astrocytes regulate T cells at multiple check points to help ensure neuroprotection and maintenance of an anti-inflammatory environment within the CNS. *“Activation of protease-activated receptor 2-mediated signaling by mast cell tryptase modulates cytokine production in primary cultured astrocytes”* by X. Zeng et al. is an original article that examined the role of tryptase, which is a protease-activated receptor (PAR)-2 agonist. Tryptase is produced by mast cells and activates the PAR-2 receptor. The authors show evidence that tryptase increased IL-6 and TNF-a production by primary astrocytes. Antagonism of PAR-2 prevented the induction of these cytokines by primary astrocytes. Moreover, the authors outlined two different signaling pathways by which tryptase promotes IL-6 (PI3-kinase dependent) and TNF in astrocytes (P38/JNK-kinase dependent). Taken together this data highlight a way in which peripheral immune cells (i.e., mast cells) can functionally communicate with astrocytes and potentially influence inflammatory responses within the CNS. *“ATP is required and advances cytokine-induced gap junction formation in microglia in vitro”* by P. J. Sáez et al. reports on the effects of ATP on microglial cells. In this research article the authors use an in vitro approach to study the function of microglial hemichannels and gap junction channels, and their data support the idea that extracellular ATP affects the cellular communication between microglia through autocrine and paracrine mechanisms. *“Possible involvement of TLRs and hemichannels in stress-induced CNS dysfunction via mastocytes, and glia activation”* by A. Aguirre et al. reviews the involvement of a TLR-mediated pathway to control neuroinflammatory responses within the CNS. The authors suggest that mastocytes and glial cells sense the inflammatory tone, via TLR activation, driving the release of inflammatory mediators and further amplification of neural damage.

A significant part of this special issue is devoted to the study of multiple sclerosis, as a paradigm of immune-to-brain communication. *“Persistent inflammation in the CNS during chronic EAE despite local absence of IL-17 production”* by S. F. G. Zorzella-Pezavento et al. describes a study that investigates the role of proinflammatory cytokines in immune infiltrates during the acute and chronic diseases stages of experimental allergic encephalitis (EAE). The results show that the levels of IFN-g are elevated in the periphery and CNS in both the acute and chronic stages of disease. In contrast, IL-17 is only found in the CNS at the acute stages; the authors suggest that this is due to a decline in Treg cells in the CNS at the chronic phase. The study did not investigate the role of myeloid cells, which may be an alternative explanation for persisting infiltrates in the CNS, despite the lack of IL-17. *“Local overexpression of interleukin-11 in the central nervous system limits demyelination and enhances remyelination”* by A. Maheshwari et al. provides evidence that IL-11 promotes remyelination in the cuprizone model of demyelination associated with reduced microglial activation. It remains to know whether the effect of IL-11 on myelination is direct or indirect via microglia/macrophages. *“Growth arrest specific gene 6 protein concentration in cerebrospinal fluid correlates with relapse severity in multiple sclerosis”* by P. P. Sainaghi et al. reports a clinical study that shows a correlation of Gas-6 in CSF with the severity of relapse in relapsing-remitting multiple sclerosis patients. However, the Gas-6 levels in CSF were not predictive for the long-term outcomes.* “Regulation of immune cell infiltration into the CNS by regional neural inputs explained by the gate theory” *by Y. Arima et al. reviews the modes of infiltration of immune cells into the CNS parenchyma. In particular, the authors propose the “gate theory” as an alternative explanation for the control of cell extravasation into the CNS in pathological conditions such as multiple sclerosis.

Some articles focus on the study of the neuroimmune component of neurodegenerative diseases such as Alzheimer's or Parkinson's, or other brain diseases. *“Role of scavenger receptors in glia-mediated neuroinflammatory response associated with Alzheimer's disease”* by F. Cornejo and R. von Bernhardi is a detailed review about the inflammatory mechanisms mediated by microglia and astroglia on the pathological events in the Alzheimer's disease, emphasizing the relevance of A scavenger receptors for Ab clearance.* “Role of neuroinflammation in adult neurogenesis and Alzheimer disease: therapeutic approaches”* by A. Fuster-Matanzo et al. reviews the influence of the neuroinflammatory component of Alzheimer's disease over the regulation of adult neurogenesis, aiming at summarizing the reported effects of several inflammatory mediators and finding new avenues into potential therapeutic interventions.* “Cellular and molecular mediators of neuroinflammation in the pathogenesis of Parkinson's disease” *by S. V. More et al. reviews the current evidence supporting a detrimental role of microglial cells during the pathogenesis of Parkinson's disease. Current evidence from human studies highlights the neurotoxic activity of diverse inflammatory mediators or infiltrated immune cells in Parkinson's disease, although further experimental evidence is needed to fully understand the neuroinflammatory reaction in this pathology. *“MMP-3 contributes to nigrostriatal dopaminergic neuronal loss, BBB damage, and neuroinflammation in an MPTP mouse model of Parkinson's disease”* by Y. C. Chung et al. is an original research article that examined the role of metalloproteases 3 (MMP3) in a mouse model of Parkinson disease. These authors provide novel evidence that matrix MMP3 plays a key role in increased leukocyte infiltration and dopaminergic cell loss after MPTP. For instance, MMP3 knockout mice had attenuated damage to the blood brain barrier (BBB), reduced infiltration of ED-1+ and CD3+ leukocytes, and reduced neuronal loss. Moreover, motor function was maintained in the MMP3 knockout mice after MPTP injection compared to the wildtype MPTP-injected mice. Thus, MMP3-mediated breakdown of the BBB and enhanced leukocyte infiltration into the brain play a significant role in nigrostriatal dopaminergic neuronal loss after MPTP. *“Are onconeural antibodies a clinical phenomenology in paraneoplastic limbic encephalitis?” *by H. Zhang et al. is a detailed review about the role of brain-specific antibodies in neurological diseases and in particular those associated with peripheral malignancies. *“The causative pathogen determines the inflammatory profile in cerebrospinal fluid and outcome in patients with bacterial meningitis”* by D. Grandgirad et al. is a clinical study that examined cerebrospinal fluid (CSF) from patients with meningitis caused by one of three different pathogens (*Streptococcus pneumonia, Neisseria mengitidis*, or *Haemophilus influenza*). The authors' findings indicate that pneumococcal meningitis was associated with high levels of inflammatory mediators in the CSF including IFN-*γ*, MCP-1, and MM9. Furthermore, the higher rates of fatal outcome in patients with pneumococcal meningitis were associated with correspondingly high levels of proinflammatory cytokines in the CSF including TNF-*α*, IL-1*β*, and IL-6. Overall, patients with pneumococcal meningitis had an elevated CNS inflammatory response and higher mortality rate than patients with either *Neisseria mengitidis* or *Haemophilus influenza*.* “Prognostic value of inflammatory mediators in 1-year outcome of acute ischemic stroke with middle cerebral artery stenosis”* by X. Gong et al. is a clinical study reporting the prognostic value of diverse inflammatory mediators in ischemic stroke. Elevated hs-CRP was found to predict 1-year poor outcome in acute stroke. The combination of increased hs-CRP, WBC, or HCY had a stronger predictive value in poor outcome than individual elevated mediator.

